# GRWD1 inhibits nucleolar stress and reduces the sensitivity of hepatocellular carcinoma to oxaliplatin

**DOI:** 10.1016/j.gendis.2025.101725

**Published:** 2025-06-18

**Authors:** Yuanyuan Li, Yumeng Wu, Shenghuan Zuo, Wenjing Zhao, Jibin Liu, Yilang Wang, Xiubing Zhang, Jian Xu, Feng Sun, Dianzheng Zhang, Shudong Zhu, Aiguo Shen

**Affiliations:** a Cancer Research Center Nantong, Nantong Tumor Hospital, Affiliated Tumor Hospital of Nantong University, Nantong, Jiangsu 226321, China; b Internal Medicine Department, Affiliated Maternity and Child Healthcare Hospital of Nantong University, Nantong, Jiangsu 226019, China; c Department of Medical Oncology, Nantong Second People's Affiliated Hospital of Nantong University, Nantong, Jiangsu 226002, China; d Department of Bio-medical Sciences, Philadelphia College of Osteopathic Medicine, Philadelphia, PA 19131, USA

Liver cancer is the fourth leading cause of cancer-related deaths worldwide. Hepatocellular carcinoma (HCC) constitutes about 90% of liver cancer cases. Most HCC patients are not diagnosed until the disease has progressed to intermediate or advanced stages, rendering effective treatments unfeasible.^1^ Transarterial chemoembolization (TACE) is a standard treatment for intermediate-stage HCC. However, approximately 50% of patients develop resistance to TACE, presenting a significant clinical challenge.^2^ Oxaliplatin (OXA), commonly used in TACE, primarily exerts its cytotoxic effects through nucleolar stress pathways.^3^ However, resistance to OXA often develops. Although several resistance mechanisms, including the prostate cancer gene expression marker 1 (PCGEM1)/miR-129-5p/ETS variant 1 (ETV1) and lysine-specific demethylase 1 (LSD1)/long intergenic non-protein coding RNA 1134 (LINC01134)/specificity protein 1 (SP1)/p62 pathways, have been identified recently, most investigations have been limited to *in vitro* studies. Here, we report glutamate-rich WD repeat containing 1 (GRWD1) as a novel mediator of TACE resistance in HCC. Mechanistically, GRWD1 reduces OXA sensitivity by competing with mouse double minute 2 (MDM2) for nucleophosmin 1 (NPM1) binding, thereby disrupting NPM1-mediated p53 stabilization and inhibiting nucleolar stress responses. Furthermore, GRWD1 expression correlates with TNM staging and shows promise as an independent prognostic biomarker for HCC. These findings provide new insights into both the diagnosis and treatment of HCC.

We first analyzed the TACE resistance transcriptome dataset (GSE104580) and identified significant dysregulation of nucleolar stress-associated pathways, including MYC, hypoxia, ultraviolet response, and p53 signaling, accompanied by altered expression of stress-responsive tumor suppressors such as cyclin-dependent kinase inhibitor 1A (CDKN1A), phosphatase and tensin homolog (PTEN), fatty acid synthase (FAS), and etoposide-induced protein 2.4 (EI24) (Fig. S1A, B). Gene Ontology (GO) and Kyoto Encyclopedia of Genes and Genomes (KEGG) pathway analyses demonstrated enrichment in ribosome biogenesis, cell cycle regulation, apoptotic pathways, and nuclear functions (Fig. S1C, D), suggesting that TACE resistance may stem from impaired nucleolar stress responses. To identify key molecular regulators at the intersection of nucleolar stress and TACE resistance in HCC, we performed weighted gene coexpression network analysis (WGCNA) on a TACE resistance dataset (23,520) and nucleolar stress dataset (2548) and identified 2206 overlapping gene products (Fig. S2A). Integrating TACE resistance-associated gene modules with the interactome of NPM1, a well-established marker of nucleolar stress,^4^ we pinpointed GRWD1 as a central hub gene within the nucleolar stress response network (Fig. S2).

To investigate GRWD's role in nucleolar stress response, we first studied its dynamic relationship with NPM1. OXA treatment induced time-dependent increases in GRWD1 and NPM1 expression in HCC cell lines, peaking at 24 h (Fig. 1A; Fig. S3A). Immunofluorescence assay showed both proteins redistributed from the nucleolus to the nucleoplasm following OXA exposure, with unchanged colocalization, suggesting a potential functional interaction between them during this process (Fig. 1B; Fig. S3B, C). We first evaluated GRWD1's clinical significance through a comprehensive analysis of The Cancer Genome Atlas (TCGA) datasets. The analysis demonstrated significant up-regulation of GRWD1 across multiple cancer types, especially in HCC tissues compared with adjacent normal liver tissues (Fig. S4). Using both publicly available datasets (*n* = 50 paired samples, *p* < 0.005) and our institutional cohort of matched HCC-normal tissue pairs (*n* = 8), we confirmed GRWD1 overexpression in HCC specimens (Fig. 1C).

**Figure 1:**
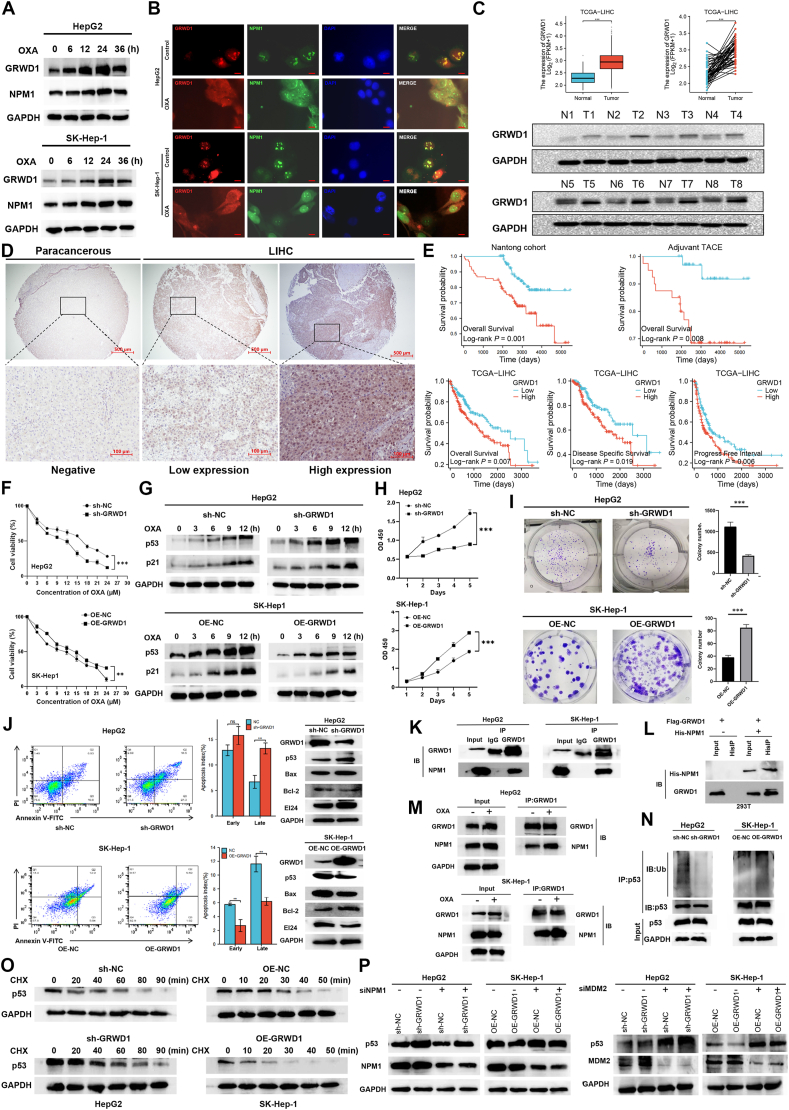
GRWD1 inhibits nucleolar stress and reduces the sensitivity of HCC to OXA. **(A)** Protein expression levels of GRWD1 and NPM1 after OXA treatment in HepG2 and SK-hep1 cells. Cells were treated with 15 μM OXA, and the protein levels of GRWD1, NPM1, and GAPDH (loading control) were detected at 0, 6, 12, 24, and 36 h. **(B)** Immunofluorescence detection of cellular localization of GRWD1 (red) and NPM1 (green) in HepG2 and SK-hep1 cells after treatment with 20 μM OXA for 12 h. Nuclei were stained with DAPI (blue), and the merge represents the overlay of all three channels. Upper panel of each cell line: untreated control; lower panel: OXA-treated. Bar = 10 μm. **(C)** Expression of GRWD1 in liver cancer samples in the TCGA database and paired tissue samples. Top left: GRWD1 mRNA expression levels in liver cancer tissues (*n* = 374) and paracancerous tissues (*n* = 50) from the TCGA database, presented as Log_2_(FPKM+1). Top right: Paired analysis of GRWD1 mRNA expression in 50 matched liver cancer and paracancerous tissue samples from the TCGA database. Bottom: Western blotting analysis of GRWD1 protein levels in 8 paired liver cancer and paracancerous tissue samples, with GAPDH serving as the loading control. **(D)** Typical examples of GRWD1 immunohistochemistry in liver cancer (LIHC) (middle: low expression; right: high expression) and paracancerous tissues (left), with scales representing 500 and 100 μm. **(E)** Top left: OS analysis of 190 HCC patients from the Nantong cohort. Patients with high GRWD1 expression had significantly worse OS than those with low expression. Top right: OS analysis of 74 HCC patients who received adjuvant TACE after HCC resection in the Nantong cohort. Patients with high GRWD1 expression exhibited significantly worse OS compared with those with low expression. Bottom: OS, DSS, and PFI of HCC patients in the TCGA database. Patients with high GRWD1 expression showed significantly worse OS, DSS, and PFI compared with those with low GRWD1 expression. **(F)** Viability of HepG2 and SK-Hep1 cell lines after treatment with different concentrations of GRWD1 and OXA (0–24 μM). **(G)** p53 and p21 protein levels were measured via western blotting after cells were treated with OXA (15 μM) for different durations, with GRWD1 either knocked down or overexpressed. **(H)** Changes in cell proliferation (CCK-8 cell proliferation assay) after GRWD1 was knocked down in HepG2 cells or overexpressed in SK-Hep-1 cells. **(I)** Changes in colony formation after the knockdown or overexpression of GRWD1 in HCC cell lines. **(J)** Cells with GRWD1 overexpression or knockdown were treated with 20 μM OXA for 12 h, followed by apoptosis analyzed by flow cytometry using annexin V-FITC/PI staining (left panels) and western blotting analysis of apoptosis-related proteins (right panels). **(K)** The interaction between endogenous GRWD1 and NPM1 in HepG2 and SK-Hep-1 cells analyzed via co-immunoprecipitation. **(L)** The interaction between exogenous GRWD1 and NPM1 in 293T cells analyzed via co-immunoprecipitation. **(M)** The interaction between GRWD1 and NPM1 in HepG2 and SK-Hep-1 cells before and after OXA (20 μM) treatment for 12 h, analyzed via co-immunoprecipitation. **(N)** Ubiquitination of the p53 protein in GRWD1-silenced/overexpressing cells after treatment with MG132 (20 μM) for 4 h, examined via immunoprecipitation with an anti-p53 antibody. **(O)** Western blotting analysis of p53 levels in GRWD1-silenced HepG2 cells and SK-Hep-1 cells overexpressing GRWD1 treated with cycloheximide (CHX) (100 μg/mL) at specified time intervals. **(P)** Western blotting analysis of p53 protein levels in GRWD1-intervened HepG2 and SK-Hep-1 cells, following NPM1 knockdown (left panels) or MDM2 knockdown (right panels). GAPDH served as the internal control. GRWD1, glutamate-rich WD repeat containing 1; HCC, hepatocellular carcinoma; OXA, oxaliplatin; MDM2, mouse double minute 2; NPM1, nucleophosmin 1; GAPDH, glyceraldehyde-3-phosphate dehydrogenase; TACE, transarterial chemoembolization; DSS, disease-specific survival; PFI, progression-free interval; OS, overall survival.

To determine the clinical significance of GRWD1 in HCC, we analyzed its expression in a tissue microarray of 190 HCC cases using immunohistochemistry (Fig. 1D). Based on GRWD1 expression levels, the cases were stratified into high (*n* = 88) and low (*n* = 102) expression groups (Table S1). GRWD1 expression was significantly correlated with established prognostic indicators, including Child-Pugh grade (*p* = 0.008) and TNM stage (*p* = 0.015) (Table S1). The Kaplan–Meier survival analysis revealed that elevated GRWD1 expression was significantly associated with poor overall survival. This association was particularly evident in a subgroup of 74 patients who underwent postoperative TACE, with findings validated in the TCGA cohort (Fig. 1E). Univariate analysis revealed tumor multiplicity (*p* < 0.001), Child-Pugh grade (*p* = 0.046), TNM stage (*p* < 0.001), and GRWD1 expression (*p* = 0.005) as significant prognostic factors of survival in HCC patients. Multivariate analysis confirmed tumor multiplicity (*p* = 0.013), TNM stage (*p* < 0.001), and GRWD1 expression (*p* = 0.021) as independent predictors in the patients. In the TACE-treated subgroup, while age, tumor multiplicity, and TNM stage showed prognostic value consistently, GRWD1's effect was not statistically significant, likely due to limited sample size (Table S2, S3).

To elucidate the mechanisms of GRWD1-mediated chemoresistance, we established cell lines with GRWD1 knockdown and overexpression and evaluated their sensitivity to OXA. GRWD1 knockdown significantly reduced the IC50 of OXA in HepG2 and Huh7 cells, whereas overexpression increased OXA resistance in SK-Hep-1 and Hep3B cells (Fig. 1F; Fig. S5A–S5E). Notably, GRWD1's effect on chemosensitivity correlated with p53 status: cells with wild-type p53 (HepG2 and SK-Hep-1) exhibited more pronounced responses compared with those with mutated (Huh7) or deleted (Hep3B) p53. Since nucleolar stress releases NPM1 to stabilize p53,^5^ we examined the temporal dynamics of p53 and its downstream target, p21, following OXA treatment (Fig. S5F). Expression of both proteins increased in a time-dependent manner. GRWD1 overexpression markedly suppressed the levels of p53 and p21 in SK-Hep-1 cells (p53-wild-type), with minimal effect on Hep3B cells (p53-null). Conversely, GRWD1 depletion enhanced p53 and p21 expression in HepG2 cells (p53-wild-type), but had no effect on Huh7 cells (p53-mutant) (Fig. 1G; Fig. S5G).

We further characterized the oncogenic properties of GRWD1. In HepG2 cells, silencing GRWD1 significantly suppressed both proliferation and colony formation; in contrast, Huh7 cells showed only a minimal response. Consistently, GRWD1 overexpression markedly enhanced the proliferation and colony formation of SK-Hep-1 cells, though the effects were attenuated in Hep3B cells (Fig. 1H I; Fig. S6). We also investigated the role of GRWD1 in OXA-induced apoptosis. Our results indicate that GRWD1 suppresses OXA-induced apoptosis, especially in cells with wild-type p53 (Fig. 1J; Fig. S7A, B). Western blotting analysis further revealed that GRWD1 down-regulated the expression of pro-apoptotic genes, such as p53, Bax, and EI24, while up-regulating the expression of the anti-apoptotic gene B-cell lymphoma 2 (Bcl-2) (Fig. 1J; Fig. S7). This regulatory effect also suggested p53 dependence, as HepG2 and SK-Hep-1 cells (with wild-type p53) exhibited a stronger response compared with Huh7 (p53 mutant) and Hep3B (p53 deficient) cells.

Since we have established that GRWD1 plays a more prominent functional role in p53-wild-type cells, we subsequently focused our mechanistic studies on HepG2 and SK-Hep-1 cells. Based on our observations of GRWD1-NPM1 colocalization, we confirmed their physical interaction by coimmunoprecipitation of both endogenous and exogenous proteins, which was significantly enhanced following OXA treatment (Fig. 1K–M). Previous studies have demonstrated that after nucleolar stress is triggered, NPM1 translocates from the nucleolus to the nucleoplasm and interacts with MDM2, inhibiting MDM2-mediated ubiquitination and degradation of p53. We found that GRWD1 promoted p53 ubiquitination, thereby reducing its stability (Fig. 1N, O; Fig. S8A, B). NPM1 depletion enhanced GRWD1-mediated p53 instability, while MDM2 knockdown attenuated this effect (Fig. 1P), further elucidating the regulatory mechanism. Lastly, our *in vivo* studies also support the notion that GRWD1 plays a significant role in the sensitivity of HCC to OXA treatment (Fig. S8C). In clinical samples, the protein level of GRWD1 was negatively correlated with that of p53 (Fig. S8D), which is consistent with our findings.

In summary, we identified GRWD1 as a novel prognostic marker in HCC patients undergoing postoperative chemotherapy. Elevated GRWD1 expression is associated with poor clinical outcomes. Mechanistically, our study reveals that GRWD1 promotes chemoresistance by sequestering NPM1. This sequestration disrupts the NPM1-MDM2 interaction, thereby enhancing MDM2-mediated degradation of p53. These findings not only establish GRWD1 as a potential prognostic indicator but also suggest it as a promising therapeutic target for overcoming chemoresistance in HCC.

## CRediT authorship contribution statement

**Yuanyuan Li:** Writing – original draft, Software, Methodology, Investigation, Formal analysis, Data curation. **Yumeng Wu:** Writing – review & editing, Supervision, Software, Methodology, Investigation, Formal analysis, Data curation. **Shenghuan Zuo:** Data curation, Formal analysis, Writing – review & editing. **Wenjing Zhao:** Data curation, Formal analysis, Writing – review & editing. **Jibin Liu:** Resources, Formal analysis. **Yilang Wang:** Funding acquisition, Formal analysis, Data curation. **Xiubing Zhang:** Funding acquisition, Formal analysis. **Jian Xu:** Formal analysis, Data curation. **Feng Sun:** Investigation, Funding acquisition, Formal analysis. **Dianzheng Zhang:** Writing – review & editing. **Shudong Zhu:** Writing – review & editing, Writing – original draft, Supervision. **Aiguo Shen:** Supervision, Conceptualization.

## Ethics declaration

Ethics approval was obtained from the Institutional Review Board (IRB) committee of Nantong Tumor Hospital (Approval Number: 2023-009). All patients provided written informed consent prior to their participation in the study, ensuring compliance with ethical standards and patient autonomy throughout the research process.

## Funding

This research was funded by grants from the Nantong Science and Technology Foundation (Jiangsu, China) (No. JC22022011, JC22022084), the Scientific Research Project of 10.13039/100017962Health Commission of Jiangsu Province, China (No. M2022091), the General Project of Nantong Health Commission (Jiangsu, China) (No. MS2022043), and the Clinical Special Research Project of Nantong University (Jiangsu, China) (No. 2022JZ013).

## Conflict of interests

The authors declared no competing interests.

## Figures and Tables

**figs1:**
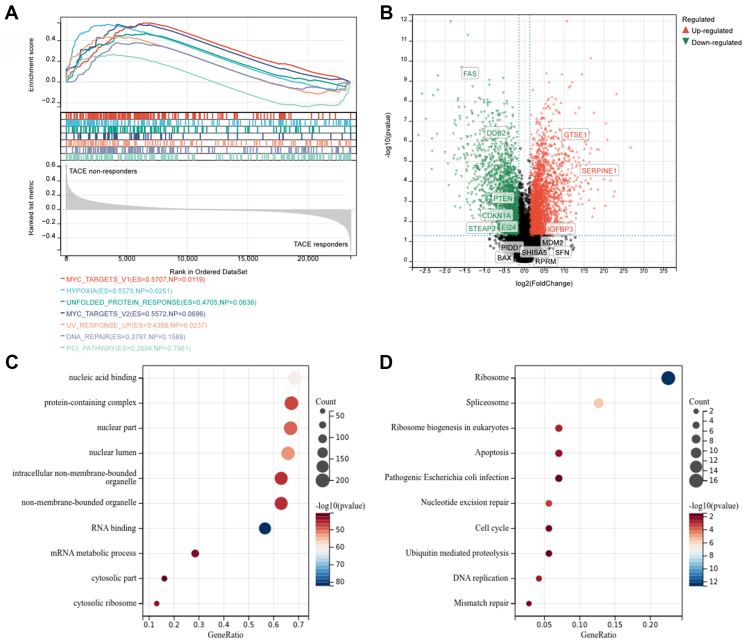


**figs2:**
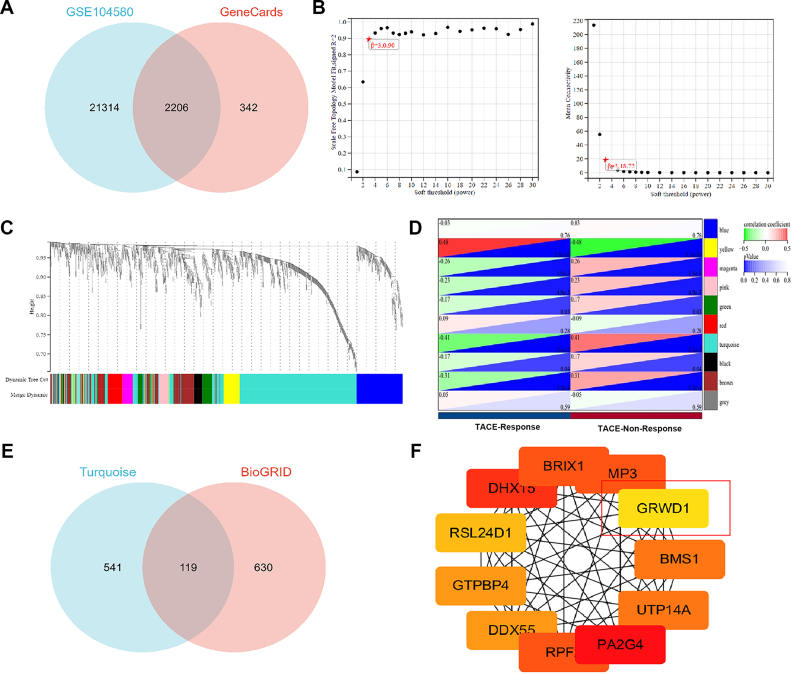


**figs3:**
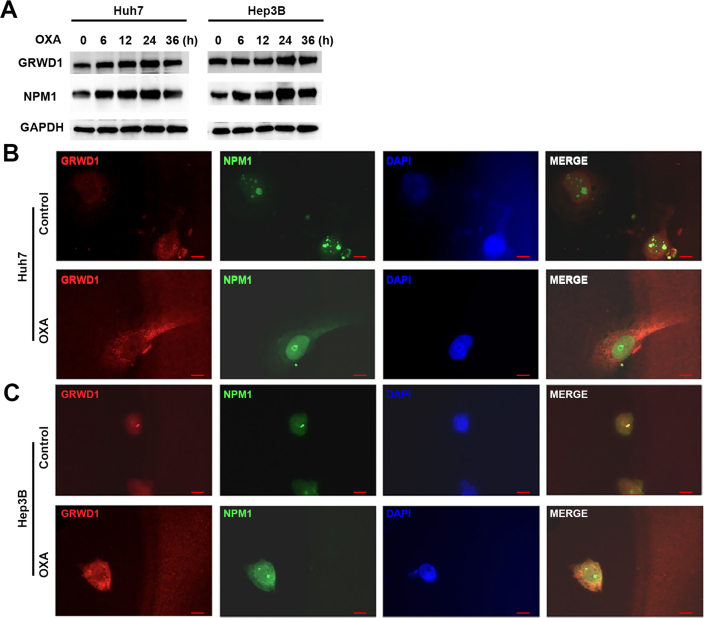


**figs4:**
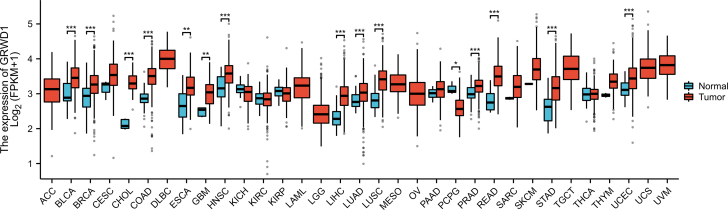


**figs5:**
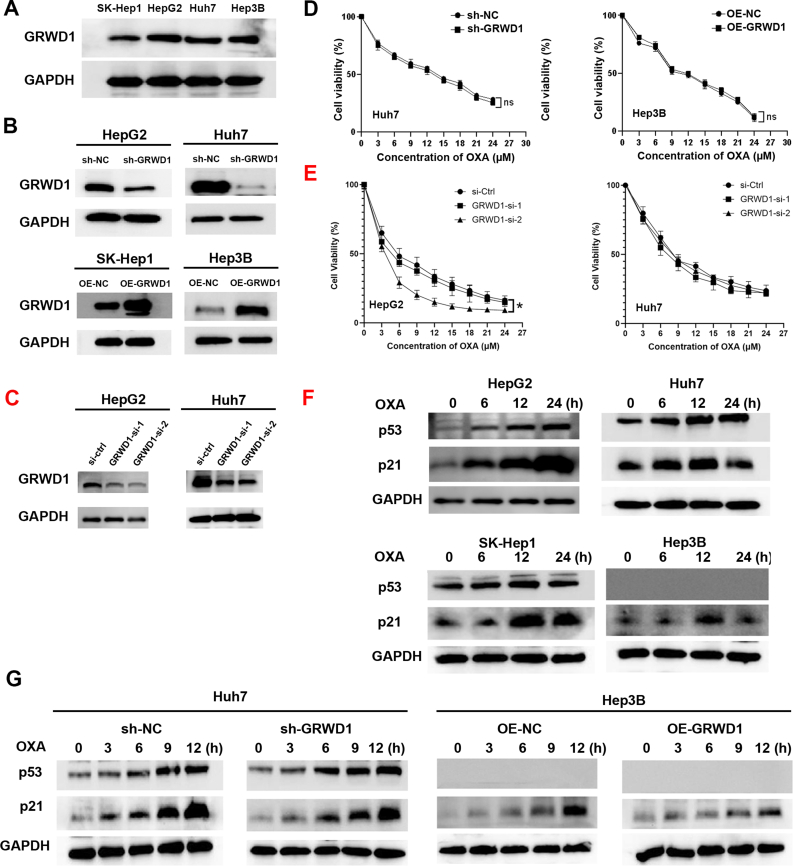


**figs6:**
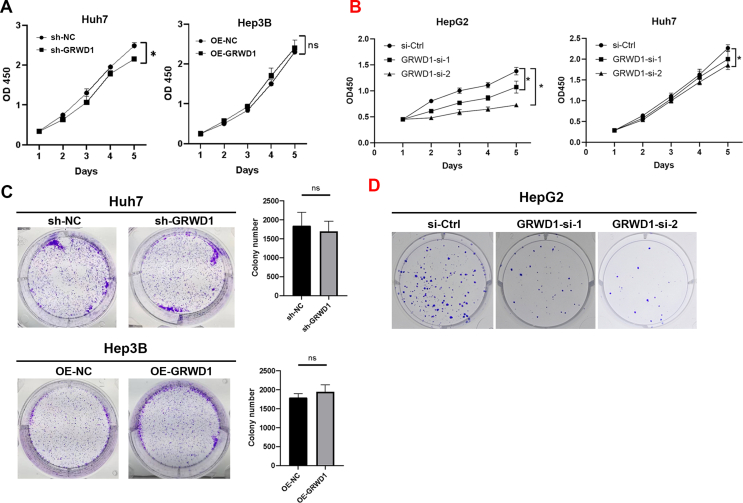


**figs7:**
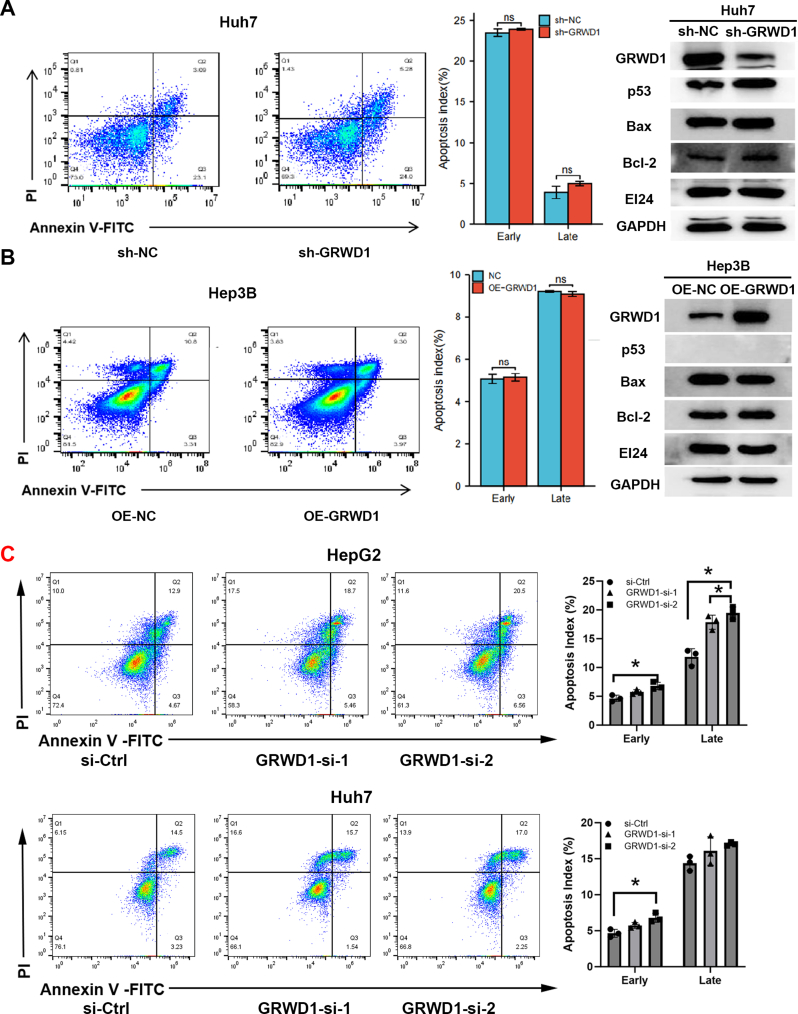


**figs8:**
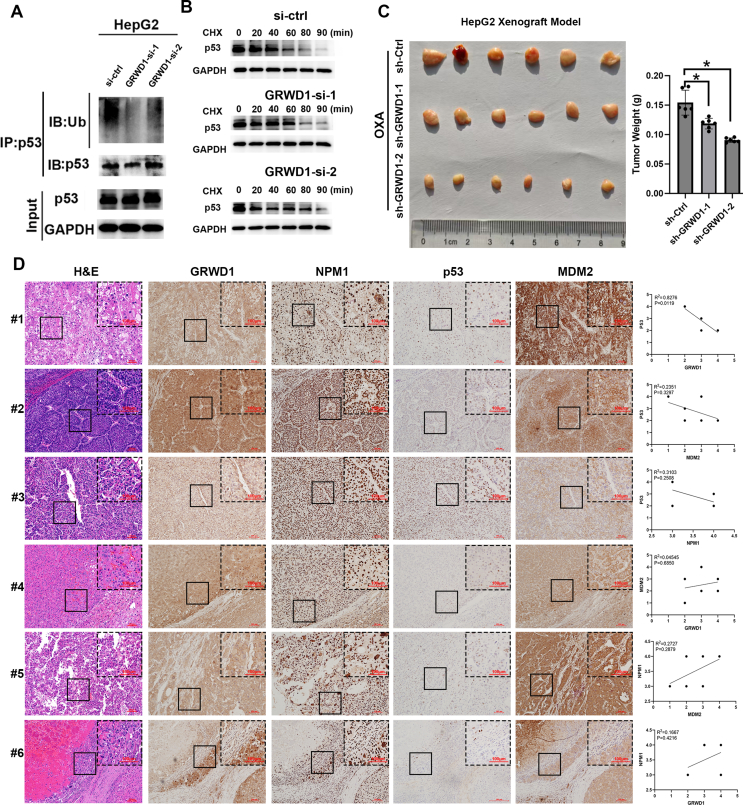


## References

[1] Llovet J.M., Kelley R.K., Villanueva A. (2021). Hepatocellular carcinoma. Nat Rev Dis Primers.

[2] Yang C., Luo Y.G., Yang H.C., Yao Z.H., Li X. (2022). Effects of early TACE refractoriness on survival in patients with hepatocellular carcinoma: a real-world study. J Hepatocell Carcinoma.

[3] Sutton E.C., McDevitt C.E., Prochnau J.Y. (2019). Nucleolar stress induction by oxaliplatin and derivatives. J Am Chem Soc.

[4] Lafita-Navarro M.C., Conacci-Sorrell M. (2023). Nucleolar stress: from development to cancer. Semin Cell Dev Biol.

[5] Yang K., Wang M., Zhao Y. (2016). A redox mechanism underlying nucleolar stress sensing by nucleophosmin. Nat Commun.

