# Supplementary material for: GRWD1 inhibits nucleolar stress and reduces the sensitivity of hepatocellular carcinoma to oxaliplatin
Source: Genes Dis. 2025 Jun 18;13(2):101725. doi: 10.1016/j.gendis.2025.101725 (PMC12607029; doi:10.1016/j.gendis.2025.101725)
Supplement: Multimedia component 1 [file mmc1.docx]

**Materials and Methods**

**Materials**

Rabbit anti-human GRWD1 monoclonal antibody, murine anti-human p53 monoclonal antibody, murine anti-human NPM1 monoclonal antibody, rabbit anti-human EI24 polyclonal antibody, murine anti-human MDM2 monoclonal antibody, and rabbit anti-human ubiquitin polyclonal antibody were obtained from Proteintech, Inc. Mouse anti-human GAPDH monoclonal antibody, rabbit anti-human p21 monoclonal antibody, rabbit anti-human Bax monoclonal antibody, and murine anti-human Bcl-2 monoclonal antibody were obtained from Cell Signaling Technology.

Ethics approval was obtained from the IRB of Tumor Hospital Nantong for a total of 190 cases. The cases were from January 1, 2007, to December 31, 2016, with follow-up until June 16, 2022. The selection of tissue samples from all the selected patients was based on the following criteria: (1) all the patients had imaging diagnosis data; (2) all the liver cancer tissue samples had clear postoperative pathological diagnoses; (3) none of the patients had received chemoradiotherapy, targeted therapy, interventional therapy, or immunotherapy before surgery; (4) histological grading of all the patients was performed according to the WHO (2008) standards; (5) detailed and complete clinicopathological data were available for all liver cancer patients enrolled. These tissue samples were collected and made into tissue chips.

**Bioinformatics analysis of public tumor databases**

The GSE104580 transcriptomic dataset, which includes samples that are responsive and non-responsive to TACE for HCC, was downloaded from the Gene Expression Omnibus (GEO, a public functional genomics data repository, available at www.ncbi.nlm.nih.gov/geo/). Additionally, mRNA sequences and corresponding clinical data for HCC were sourced from the TCGA database (The Cancer Genome Atlas, https://portal.gdc.cancer.gov/repository), which provides comprehensive genomic data on various cancers.

To investigate the relationship between TACE resistance and nucleolar stress-related pathways, Gene Set Enrichment Analysis (GSEA) was conducted using the software available at http://www.broadinstitute.org/gsea. GSEA is a computational method that determines whether an a priori defined set of genes shows statistically significant, concordant differences between two biological states (e.g., TACE responsive vs. non-responsive). The signaling pathways and cellular biological behaviors associated with TACE resistance were examined through KEGG (Kyoto Encyclopedia of Genes and Genomes) and GO (Gene Ontology) enrichment analyses. These analyses were performed using the OmicShare tools (www.omicshare.com/tools), which offer a suite of bioinformatics analysis functions.

Weighted gene co-expression network analysis (WGCNA) was carried out on samples from the GSE104580 dataset using the R package "WGCNA" (version 1.69). WGCNA aims to identify modules of highly correlated genes and then relate these modules to external sample traits, in this case, to identify the module gene set most strongly correlated with TACE non-responsiveness. Gene symbols for proteins interacting with NPM1 were obtained from BioGRID (https://thebiogrid.org/, a database of genetic and protein interactions).

Hub gene analysis was performed using the Stress method in Cytoscape software (version 3.7.1), a popular software platform for visualizing molecular interaction networks and biological pathways. The differential expression of GRWD1 across various cancers was analyzed using the R package "TCGAplot". Statistical analyses were performed using R 4.2.1.

**Cell lines and culture**

HepG2 and Huh7 cells [from the American Type Culture Collection (ATCC)] were cultured in DMEM supplemented with 10% fetal bovine serum (FBS), and SK-Hep-1 and Hep3B cells [from ATCC] were cultured in MEM supplemented with 10% fetal bovine serum (FBS).

**Immunofluorescence analysis**

The cells were fixed, permeabilized and immunostained with a fluorescence-labeled antibody against NPM1 or GRWD1, followed by DAPI staining, and images were acquired with a fluorescence microscope, with details as described previously (PMID: 17934522).

**Histopathological examination and immunohistochemistry (IHC)**

Histopathological examination and IHC for GRWD1, NPM1, MDM2, and p53 were conducted following the methods previously described (PMID: 33210326). To quantify the immunohistochemical results, Image analysis software (ImageJ, version 1.46a, developed by the National Institutes of Health, USA) was utilized, and the IHC Toolbox plugin was employed to assess the corresponding images. The criteria for quantifying p53 expression, as reported previously (PMID: 12046070), were as follows: Nuclear p53 expression in ≥ 10% of tumor cells was defined as aberrant overexpression. The scoring system was: < 10% = 1, 10%-30% = 2, 31%-50% = 3, and > 50% = 4. Finally, Pearson's correlation coefficient was used to calculate the correlations among the expression levels of GRWD1, NPM1, MDM2, and p53 proteins.

**Western Blot (WB)**

WB was performed as previously described (PMID: 33210326).

**Transfections**

Lentiviruses for knocking down and overexpressing GRWD1 were purchased from Shanghai Genechem Co., Ltd. Viral transduction was performed according to the manufacturer’s instructions. Cell proteins were collected 2 days after for Western blot analysis to assess the transduction efficiency. The GRWD1 overexpression plasmid and the NPM1 overexpression plasmid were purchased from Shanghai Genechem Co., Ltd. and WZ Biosciences Co., Ltd., respectively. Small interfering RNAs (siRNAs) targeting NPM1 and MDM2 were purchased from OBiO Technology Co., Ltd. The siRNAs GRWD1-si-1 and GRWD1-si-2 were synthesized by GenScript Biotech Corporation. Plasmid and siRNA transfection were performed using JetPRIME® Polyplus transfection reagent as per the manufacturer’s instructions. The culture medium was changed 5 hours after transfection. Cells were collected 48 hours after plasmid transfection or 72 hours after siRNA transfection for subsequent experiments.

The shRNA and siRNA sequences for the GRWD1, and the siRNA sequences for NPM1, and MDM2 genes are as follows:

sh-GRWD1:

5'- CGGACAGAGCUUCCUCUUATT-3' (sense);

5’- UAAGAGGAAGCUCUGUCCGTT-3’ (antisense);

sh-GRWD1-1 and si-GRWD1-1:

5'-GAUGCUUCGGAUGCACAAUUU-3'(sense);

5'-AUUGUGCAUCCGAAGCAUCAU-3'(antisense);

sh-GRWD1-2 and si-GRWD1-2:

5'-CCUCUUACACUUUACUUGUUU-3'(sense);

5'-ACAAGUAAAGUGUAAGAGGAA-3'(antisense);

siNPM1:

5'-CGAUGAUGAUGAAGAGGAUTT-3'(sense);

5'-AUCCUCUUCAUCAUCAUCGTT-3'(antisense);

siMDM2:

5’-CUGGCUCUGUGUGUAAUAATT-3' (sense);

5'-TTUUAUUACACACAGAGCCAG-3' (antisense);

**Cell proliferation assay**

Transfected or treated cells were seeded in 96-well plates at a density of 4×10^3^ cells per well. Cell viability was assessed using a CCK-8 cell counting kit.

**Cell colony formation assay**

The treated HCC cells were uniformly inoculated into 6 - well plates at the same density. After a two - week incubation period, the colonies were fixed with 4% paraformaldehyde and stained with 0.5% crystal violet.

**Apoptosis assay**

The cells were digested and then collected by centrifugation. Subsequently, they were resuspended in phosphate - buffered saline (PBS) and collected again by centrifugation.

After adding the appropriate buffers, annexin V - FITC was added in the dark, and the mixture was incubated at room temperature for 15 minutes. Next, 5 μl of propidium iodide (PI) solution was added, and the mixture was further incubated at room temperature for 5 minutes. Finally, 200 μL of buffer was added, and the sample was ready for flow cytometry analysis.

**Real-time quantitative reverse transcription polymerase chain reaction (qRT‒PCR)**

Total RNA was extracted using an RNA extraction kit, and cDNA was synthesized using a reverse transcription kit. cDNA amplification via qRT‒PCR with SYBR Green was performed on a Bio-Rad CFX96 real-time fluorescence quantitative PCR machine. The amplification conditions were as follows: initial denaturation at 95°C for 5 minutes, followed by 40 cycles of denaturation at 95°C for 10 seconds and annealing/extension at 60°C for 30 seconds. GAPDH was used as the internal control for mRNA quantification. The relative expression of the GRWD1 mRNA was calculated using the 2-ΔΔCt method. The sequences of the human primers used for the GRWD1 and GAPDH genes are as follows:

GRWD1: 5'-CCATCTTCTCCTTCGCTGGACA-3' (sense);

5’-AGGTGTCCAGAGGTGGATGTTC-3’ (antisense);

GAPDH: 5’-CATGAGAAGTATGACAACAGCCT-3’ (sense);

5’-AGTCCTTCCACGATACCAAAGT-3’ (antisense).

**Co-immunoprecipitation (Co-IP)**

Co-IP was performed as previously described (PMID: 17934522).

**Half-life and ubiquitination assays**

To conduct the half - life assay, WB was used to detect p53 levels in GRWD1-silenced or GRWD1-overexpressing cells treated with cycloheximide (CHX) at a concentration of 100 µg/ml. For the ubiquitination assay, cells were treated with 20 µM MG132 for 4 hours. Subsequently, immunoprecipitation was performed on whole-cell lysates using an anti-p53 antibody. The immunoprecipitates were then analyzed by WB with an anti-ubiquitin antibody.

***In vivo*** studies

All animals received humane care and complied with the guidelines for the use of laboratory animals established by the Animal Care and Use Committee of Nantong Tumor Hospital . HepG2 cells were trypsinized, washed twice with PBS, and then resuspended in PBS. Six-week-old male nude mice were used. Each mouse was subcutaneously inoculated in the right axilla with a 200-μL mixture containing 1×10^7^ cells and 100 μL of Matrigel. After the inoculation of HepG2 cells, the mice were given intraperitoneal injections of OXA at a dosage of 5 mg/kg twice a week. The mice were sacrificed 32 days after treatment. Tumors were then harvested, photographed, and weighed (Ma et al., 2021, *Hepatology*, PMID:34322883).

**Data processing**

Statistical analyses were performed using GraphPad Prism software version 8.0 (GraphPad Software, Inc.) and R software (version 4.2.1). Differences between groups were assessed using either the Chi-square test or Student's t-test, depending on the data characteristics. Post-operative survival was estimated using the Kaplan–Meier method, and prognostic factors were analyzed by Cox stepwise multiple regression. A P-value less than 0.05 was considered statistically significant.
