# Supplementary material for: GRWD1 inhibits nucleolar stress and reduces the sensitivity of hepatocellular carcinoma to oxaliplatin
Source: Genes Dis. 2025 Jun 18;13(2):101725. doi: 10.1016/j.gendis.2025.101725 (PMC12607029; doi:10.1016/j.gendis.2025.101725)
Supplement: Multimedia component 2 [file mmc2.docx]

**Table S1** Analysis of the correlation between GRWD1 expression levels and clinicopathological features in liver cancer tissues

| Characteristics | GRWD1 High | GRWD1 Low | *P* |
| --- | --- | --- | --- |
| All patients, n | 88 | 102 |  |
| Gender, n (%) |  |  | 0.412 |
| Male | 70 (36.8%) | 76 (40%) |  |
| Female | 18 (9.5%) | 26 (13.7) |  |
| Age, n (%) |  |  | 0.696 |
| >50 | 61 (32.1%) | 68 (35.8%) |  |
| ≤50 | 27 (14.2%) | 34 (17.9%) |  |
| Tumor size, n (%) |  |  | 0.624 |
| ≤5cm | 61 (32.1%) | 74 (38.9%) |  |
| >5cm | 27 (14.2%) | 28 (14.7%) |  |
| Tumor quantity, n (%) |  |  | 0.937 |
| Single | 72 (37.9%) | 83 (43.7） |  |
| Multiple | 16 (8.4%) | 19 (10%) |  |
| Histological grading, n (%) |  |  | 0.328 |
| Well differentiated-Moderately differentiated | 59 (31.1%) | 75 (39.5%) |  |
| Poorly differentiated | 29 (15.3%) | 27 (14.2%) |  |
| Vascular tumor thrombus, n (%) |  |  | 0.189 |
| Yes | 17 (8.9%) | 28 (14.7%) |  |
| No | 71 (37.4%) | 74 (38.9%) |  |
| Cirrhosis, n (%) |  |  | 0.227 |
| Yes | 50 (26.3%) | 49 (25.8%) |  |
| No | 38 (20%) | 53 (27.9%) |  |
| Splenomegaly, n (%) |  |  | 0.637 |
| Yes | 62 (32.6%) | 75 (39.5%) |  |
| No | 26 (13.7%) | 27 (14.2%) |  |
| AFP, n (%) |  |  | 0.686 |
| ≤25 ng/mL | 44 (23.2%) | 48 (25.3%) |  |
| ＞25 ng/mL | 44 (23.2%) | 54 (28.4%) |  |
| Hepatitis history, n (%) |  |  | 0.501 |
| Yes | 27 (14.2%) | 36 (18.9%) |  |
| No | 61 (32.1%) | 66 (34.7%) |  |
| Child-Pugh stage, n (%) |  |  | **0.008*** |
| A | 62 (32.6%) | 88 (46.3%) |  |
| B | 26 (13.7%) | 14 (7.4%) |  |
| TNM stage, n (%) |  |  | **0.015*** |
| 1-2 | 50 (26.3%) | 75 (39.5%) |  |
| 3-4 | 38 (20%) | 27 (14.2%) |  |

*P＜0.05

**Table 2** Cox Regression Analysis of Clinicopathological Parameters, GRWD1 Expression, and Survival Prognosis in HCC Patients

|  | Univariate |  | Multivariate |  |
| --- | --- | --- | --- | --- |
| Characteristics | Hazard Ratio (95% Confidence Interval) | *P* | Hazard Ratio (95% Confidence Interval) | *P* |
| Gender | 0.922 | 0.811 |  |  |
| Male VS Female | (0.470-1.806) |  |  |  |
| Age | 1.156 | 0.637 |  |  |
| (≤50 VS ＞50) | (0.629-2.126) |  |  |  |
| Tumor size | 1.426 | 0.239 |  |  |
| (≤5cm VS ＞5cm) | (0.798-2.551) |  |  |  |
| Tumor number | 3.025 | **＜0.001*** | 2.176 | **0.013*** |
| (Single VS Multiple） | (1.691-5.414) |  | (1.179-4.015) |  |
| Differentiation | 0.868 | 0.65 |  |  |
| (Poorly differentiated VS Well-Moderately differentiated） | (0.472-1.595) |  |  |  |
| Vascular tumor thrombus | 1.819 | 0.06 | 1.757 | 0.08 |
| (No VS Yes) | (0.999-3.315) |  | (0.935-3.3) |  |
| Cirrhosis | 0.697 | 0.208 |  |  |
| (No VS Yes) | (0.398-1.223) |  |  |  |
| Splenomegaly | 1.037 | 0.91 |  |  |
| (No VS Yes) | (0.556-1.933) |  |  |  |
| AFP | 1.057 | 0.848 |  |  |
| (≤25ng/mL VS ＞25ng/mL) | (0.603-1.852) |  |  |  |
| Hepatitis history | 0.929 | 0.808 |  |  |
| (No VS Yes) | (0.516-1.674) |  |  |  |
| Child-Pugh stage | 1.92 | 0.046 | 0.803 | 0.509 |
| (A VS B) | (1.043-3.531) |  | (0.418-1.541) |  |
| TNM stage | 5.572 | **＜0.001*** | 4.345 | **＜0.001*** |
| (1-2 VS 3-4) | (3.068-10.118) |  | (2.236-8.442) |  |
| GRWD1 expression | 2.273 | **0.005*** | 2.019 | **0.021*** |
| (Low VS High) | (1.271-4.067) |  | (1.11-3.673) |  |

* P＜0.05

**Table 3** Univariate and Multivariate Cox Regression Analysis of Survival in Patients Undergoing TACE after Liver Cancer Resection

| Characteristics | Univariate | *P* | Multivariate | *P* |
| --- | --- | --- | --- | --- |
|  | Hazard Ratio (95% Confidence Interval) |  | Hazard Ratio (95% Confidence Interval) |  |
| Gender | 0.797 | 0.723 |  |  |
| Male VS Female | (0.222-2.861) |  |  |  |
| Age | 7.629 | **0.009*** | 0.047 | **0.01*** |
| (≤50 VS ＞50) | (0.997-58.386) |  | (0.005-0.473) |  |
| Tumor size | 1.8 | 0.275 |  |  |
| (≤5cm VS ＞5cm) | (0.631-5.134) |  |  |  |
| Tumor number | 5.526 | **0.007*** | 4.178 | **0.035*** |
| (Single VS Multiple） | (1.834-16.649) |  | (1.107-15.774) |  |
| Differentiation | 0.493 | 0.203 |  |  |
| (Poorly differentiated VS Well-Moderately differentiated） | (0.171-1.4) |  |  |  |
| Vascular tumor thrombus | 3.826 | **0.014*** | 1.932 | 0.269 |
| (No VS Yes) | (1.325-11.049) | 0.488 | (0.601-6.214) |  |
| Cirrhosis | 1.464 | 0.494 |  |  |
| (No VS Yes) | (0.491-4.37) |  |  |  |
| Splenomegaly | 2.618 | 0.088 | 1.81 | 0.476 |
| (No VS Yes) | (0.905-7.568) |  | (0.354-9.265) |  |
| AFP | 1.706 | 0.329 |  |  |
| (≤25ng/mL VS ＞25ng/mL) | (0.571-5.091) |  |  |  |
| Hepatitis history | 1.072 | 0.9 |  |  |
| (No VS Yes) | (0.359-3.20) |  |  |  |
| Child-Pugh stage | 5.13 | **0.004*** | 1.013 | 0.987 |
| (A VS B) | (1.794-14.67) |  | (0.229-4.4) |  |
| TNM stage | 37.108 | **＜0.001*** | 47.25 | **＜0.001*** |
| (1-2 VS 3-4) | (4.805-286.547) |  | (5.488-406.803) |  |
| GRWD1 expression | 1.933 | 0.227 |  |  |
| (Low VS High) | (0.647-5.772) |  |  |  |

*P＜0.05
