# Supplementary material for: GRWD1 inhibits nucleolar stress and reduces the sensitivity of hepatocellular carcinoma to oxaliplatin
Source: Genes Dis. 2025 Jun 18;13(2):101725. doi: 10.1016/j.gendis.2025.101725 (PMC12607029; doi:10.1016/j.gendis.2025.101725)
Supplement: Multimedia component 3 [file mmc3.docx]

**Figure S1. Nucleolar stress and TACE resistance. (A)** Hallmark gene set enrichment analysis of the TACE resistance dataset GSE104580. **(B)** Differentially expressed genes in the dataset between the resistant and nonresistant groups. **(C)** KEGG pathway enrichment analysis of the differentially expressed genes. **(D)** GO enrichment analysis of the differentially expressed genes.

**Figure S2. Key genes involved in nucleolar stress (A)** A total of 2,206 overlapping genes encoding nucleolar stress proteins identified from the GSE104580 dataset (blue) and the GeneCards database (red), as shown in the middle (grey). Subsequently, weighted gene coexpression network analysis (WGCNA) was performed to cluster these genes (Figure S2B-C). **(B)** Scale-free fitting index for soft threshold powers. Left: Relationship between the soft threshold and scale-free R^2^. Right: Relationship between the soft threshold and average connectivity. **(C)** A dendrogram of the clustered overlapping genes. Different modules are marked with different colors. **(D)** Heatmaps depicting the correlations between module characteristic genes and disease states. Each cell contains a correlation coefficient and a P value. Red indicates a positive correlation, green indicates a negative correlation, and blue represents a P value. A significant difference is defined as P<0.05. The left panel shows the response to TACE, and the right panel shows the nonresponding cases. The cyan part (inside the red border) represents the module with the largest correlation coefficient in the TACE-resistance group, and a correlation coefficient of 0.41 (p < 0.001). **(E)** 119 overlapping genes (middle part) between the cyan module genes (blue part) and the NPM1-interacting proteins from the BioGRID database (red part). **(F)** The top 15 hub genes identified based on the Degree algorithm.

**Figure S3. Expression of GRWD1 and NPM1 and their nucleoplasmic translocation induced by cell stress**

**(A)** Induction of GRWD1 and NPM1 protein expression after OXA treatment. Huh7 and Hep3B cells were stimulated with OXA at a concentration of 15 µM. The cells were collected at 0, 6, 12, 24 and 36 hours post-treatment. Subsequently, WB was performed to detect GRWD1 and NPM1 protein levels, with GAPDH used as the internal protein control. **(B, C)** Immunofluorescence was employed to detect the spatial locations of GRWD1 and NPM1 after inducing cellular stress. Two cell lines were treated with OXA at a concentration of 20 µM for 12 h. In panels (B) and (C), the upper panel represents the control group, and the lower panel represents the treatment group. Scale bar = 10 μm.

**Figure S4. Upregulated expression of GRWD1 in liver cancer**

Expression of GRWD1 across various cancers in The Cancer Genome Atlas (TCGA) database was analyzed. The Wilcoxon rank - sum test was employed as the statistical method for the analysis. In the figure, asterisks (*, **, and ***) denote P - values less than 0.05, 0.01, and 0.005, respectively.

**Figure S5. GRWD1 affects the sensitivity of HCC cells to** OXA

**(A)** GRWD1 protein levels in SK-Hep-1, HepG2, Huh7, and Hep3B cells examined by WB. **(B)** GRWD1 was overexpressed in SK-Hep-1 and Hep3B cells and knocked down in HepG2 and Huh7 cells. The effect was verified by analyzing protein levels *via* WB. **(C)** Knockdown of GRWD1 in HepG2 and Huh7 cells using two independent siRNAs. (**D**) Viability of Huh7 and Hep3B cell lines after shRNA - mediated knockdown and overexpression of GRWD1, respectively, at different concentrations of OXA (0–24 µM). (**E**) Viability of HepG2 and Huh7 cells after knockdown of GRWD1 using two independent siRNAs at different concentrations of OXA (0–24 µM). (F) p53 and p21 levels were measured by WB after the cells were treated with OXA (15 µM) for various durations. **(G)** p53 and p21 protein levels in Huh7 and Hep3B cells treated with OXA (15 µM) for different durations were measured by WB, with GRWD1 either knocked down or overexpressed. GAPDH was used as an internal control. *, **, and *** indicate P < 0.05, P < 0.01, and P < 0.005, respectively, while "ns" represents no statistical significance.

**Figure S6. GRWD1 promotes the proliferation of HCC cells (A)** Changes in cell proliferation, as assessed by the CCK - 8 cell proliferation assay, following shRNA - mediated knockdown of GRWD1 in Huh7 cells or its overexpression in Hep3B cells. **(B)** Changes in cell proliferation after GRWD1 was knocked down in HepG2 and Huh7 cells using two independent siRNAs, in comparison with a control siRNA. **(C)** Changes in colony formation after shRNA - mediated knockdown or overexpression of GRWD1 in HCC cell lines. **(D)** Changes in colony formation after GRWD1 was knocked down in HepG2 cells using two independent siRNAs, when compared to a control siRNA. *, **, and *** indicate P < 0.05, P < 0.01, and P < 0.005, respectively, while "ns" represents no statistical significance.

**Figure S7. GRWD1 inhibits the apoptosis of HCC cells**

(**A, B**) Hep3B cells with GRWD1 overexpression, Huh7 cells with shRNA-mediated knockdown of GRWD1, and their corresponding control cells were treated with 20 µM OXA. The cells were collected 12 hours later for flow cytometry analysis of apoptosis and WB analysis of apoptosis-related proteins. Left, the apoptosis diagram; middle, the graphs represent the percentage of cells in early and late apoptosis; right, the WB analysis of apoptosis-related proteins. (**C**) Apoptosis was assessed by flow cytometry in HepG2 cells transfected with either a control siRNA or two independent siRNAs targeting GRWD1. The graphs show the percentage of cells in both the early and late stages of apoptosis. *, and ** indicate P < 0.05, and P < 0.01 respectively, while "ns" represents no statistical significance.

**Figure S8. *In vivo* assay and additional assays.** (A) The ubiquitination of the p53 protein in HepG2 cells where GRWD1 was knocked down by two independent siRNAs. After the cells were treated with MG132 (20 µM) for 4 hours, immunoprecipitation was performed using an anti-p53 antibody. (B) WB analysis of p53 levels in HepG2 cells in which GRWD1 was knocked down by two independent siRNAs. These cells were treated with cycloheximide (CHX) (100 µg/ml) at specific time intervals. (C) Images of resected xenograft tumors and their weights obtained 32 days after oxaliplatin (OXA) treatment in HepG2 xenograft models. Mice were injected with HepG2 cells stably expressing two independent GRWD1-knockdown shRNAs or a control shRNA, and then treated with OXA after cell injection (n = 6 per group). An asterisk (*) indicates a P - value less than 0.05. (D) Representative images of hematoxylin and eosin (H&E) staining, along with immunohistochemical staining for GRWD1, NPM1, p53, and MDM2 in HCC tissues. The scatter plots on the right illustrate the correlations between GRWD1 and p53, MDM2 and p53, NPM1 and p53, GRWD1 and MDM2, NPM1 and MDM2, and GRWD1 and NPM1. The scale bar for the inset images is 100 μm.
